# Epidemiology, Pathophysiology and Management of Atypical Femur Fractures: an Update

**DOI:** 10.1007/s11914-025-00932-3

**Published:** 2025-10-07

**Authors:** Lucy Collins, Hanh H. Nguyen, Frances Milat, Peter R. Ebeling

**Affiliations:** 1 Department of Medicine, School of Clinical Sciences, Monash University, Clayton, VIC, Australia; 2 Department of Endocrinology, Monash Health, Clayton, VIC Australia; 3 Department of Endocrinology and Diabetes, Western Health, Sunshine, VIC Australia; 4 Department of Medicine, The University of Melbourne, Parkville, VIC, Australia; 5 Hudson Institute of Medical Research, Clayton, VIC, Australia

**Keywords:** Atypical femur fracture, Anti-resorptives, Osteoporosis, Bisphosphonates, Asian ethnicity, Screening

## Abstract

**Purpose of review:**

To summarise recent publications addressing the epidemiology, pathogenesis and management of atypical femur fractures (AFFs).

**Recent findings:**

AFFs have been reported in anti-resorptive treated individuals, bisphosphonate-naïve individuals and individuals with monogenic bone diseases. The likelihood of developing an AFF increases with prolonged exposure to anti-resorptive treatment. AFF risk declines following anti-resorptive discontinuation. Asian ethnicity has emerged as an important risk factor for AFF. Although excluded from the current ASBMR AFF case definition, periprosthetic AFFs and atypical fractures at non-classical sites have been increasingly reported. Following an AFF, anti-resorptive therapy should be discontinued, surgical treatment with intramedullary nailing considered, the contralateral femur imaged, and the underlying osteoporosis addressed. Emerging evidence suggests teriparatide may aid healing in surgically managed AFFs but not in conservatively managed incomplete AFFs.

**Summary:**

AFFs remain a rare side effect of anti-resorptive treatment. Emerging areas of interest and further research include genetic and ethnic risk factors and advancements in diagnostic technologies for AFFs.

## Introduction

**Fig. 1:**
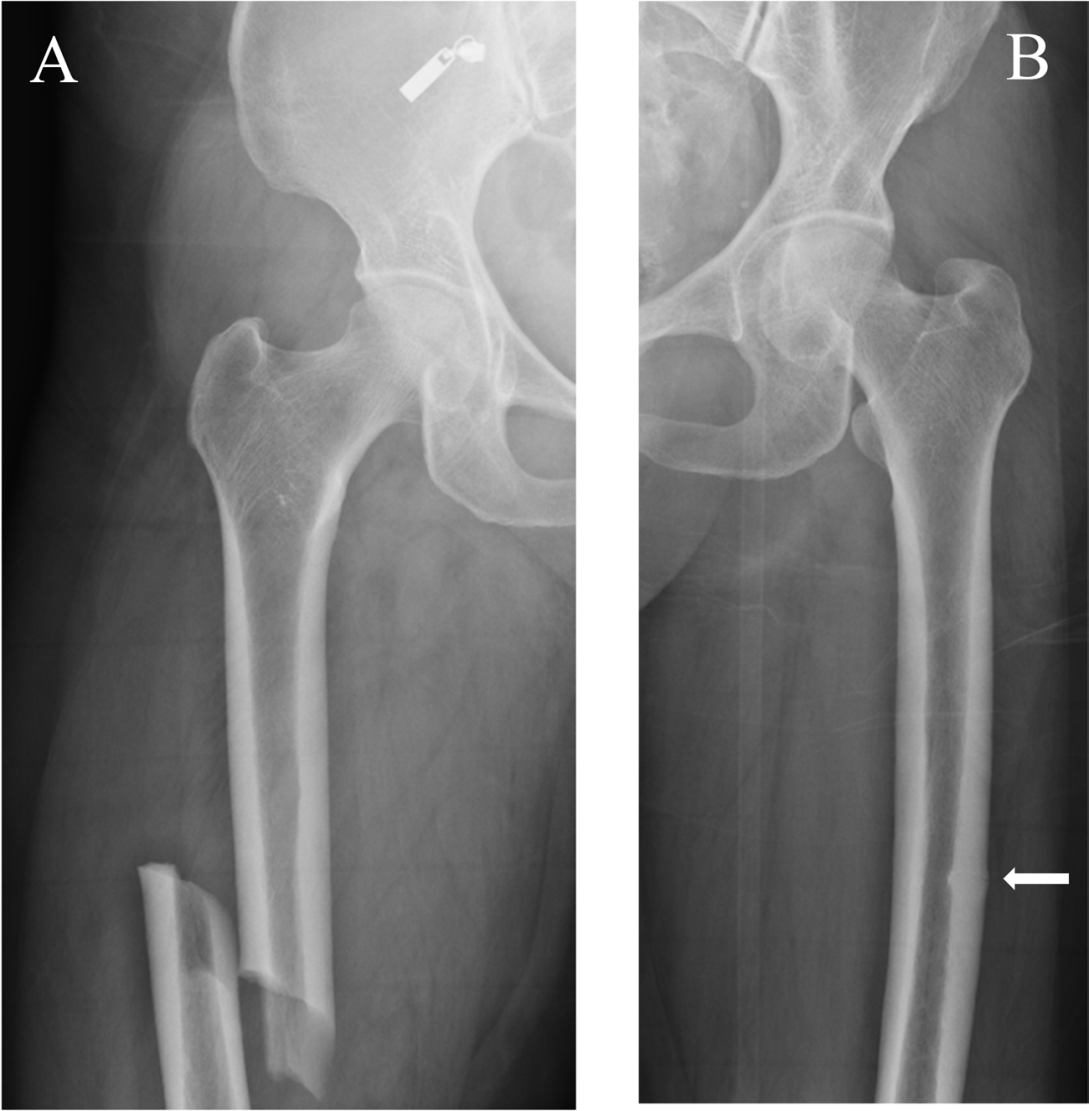
A 58-year-old woman with osteoporosis who presented with bilateral AFFs. (**A**) Anteroposterior radiograph of the right femur shows a complete AFF. (**B**) Anteroposterior radiograph of the left femur shows an incomplete AFF (white arrow)

Atypical femur fractures (AFFs) were initially described in a pivotal case series of insufficiency fractures in 2005 [1]. Nine patients, receiving long-term alendronate treatment (3–8 years), sustained a variety of atraumatic fractures, including femoral shaft fractures. The American Society for Bone and Mineral Research (ASBMR) Task Force proposed a case definition for these atypical fractures affecting the femoral diaphysis in 2010 [2]. The definition was revised in 2013 (Table 1) [3]. An updated ASBMR Task Force AFF case definition is currently in development, with publication anticipated in 2026. AFFs are classically transverse in orientation, minimally comminuted and characterized by localized thickening of the lateral cortex at the fracture site, which represents their origin as a stress fracture. These radiographic features distinguish them from typical osteoporotic femoral fractures, and they may be complete or incomplete (Fig. 1).

**Table 1:** ASBMR task force revised case definition of AFFs [3] To satisfy the case definition of AFF, the fracture must be located along the femoral diaphysis from just distal to the lesser trochanter to just proximal to the supracondylar flare. In addition, at least four of five Major Features must be present. None of the Minor Features is required but have sometimes been associated with these fractures

Major features	
The fracture is associated with minimal or no trauma, as in a fall from a standing height or less	
The fracture line originates at the lateral cortex and is substantially transverse in its orientation, although it may become oblique as it progresses medially across the femur	
Complete fractures extend through both cortices and may be associated with a medial spike; incomplete fractures involve only the lateral cortex	
The fracture is noncomminuted or minimally comminuted	
Localized periosteal or endosteal thickening of the lateral cortex is present at the fracture site (“beaking” or “flaring”)	
Minor features	
Generalised increase in cortical thickness of the femoral diaphysis	
Unilateral or bilateral prodromal symptoms such as dull or aching pain in the groin or thigh	
Bilateral incomplete or complete femoral diaphysis fractures	
"Delayed fracture healing”

AFFs have been reported in anti-resorptive treated individuals, families, bisphosphonate-naïve individuals and individuals with monogenic bone diseases. Whilst anti-resorptive medications, bisphosphonates and denosumab, are effective in the management of osteoporosis, evidence has demonstrated that the risk of sustaining an AFF increases with longer duration of treatment [4, 5]. Despite this, the overall incidence of AFF is low [5]. Furthermore, the occurrence of AFFs in families, untreated individuals and those with an underlying monogenic bone disease has sparked speculation about a genetic contribution to AFF susceptibility [6, 7]. Additional postulated risk factors include younger age, Asian ethnicity, higher body-mass index (BMI), rheumatoid arthritis, varus proximal femoral geometry, lateral femoral bowing and the use of systemic glucocorticoids [3, 8, 9].

This review focuses on recent publications addressing the epidemiology, pathogenesis and management of AFFs. Emerging areas of interest, including genetic and ethnic risk factors and advancements in diagnostic technologies for AFFs, are explored.

### Update on Epidemiology

In order to ascertain an accurate estimation of AFF incidence and the increase in risk attributable to bisphosphonate therapy, longitudinal cohort studies adjudicating AFFs based on the ASBMR AFF case definition are critical [3, 5, 10]. Numerous previous studies have used International Classification of Disease (ICD) codes to diagnose subtrochanteric and femoral shaft (ST/FS) femoral fractures. However, only a small proportion of ST/FS are AFFs [5]. In recent years, key limitations have been addressed through the publication of several large longitudinal cohort studies that use ASBMR-adjudicated definitions of AFF. These studies offer substantial improvement to our understanding of the relationship between bisphosphonate exposure and AFF risk, the impact of drug holidays on AFF risk and the relationship of numerous clinical factors to AFF development [4, 11].

#### Duration of Bisphosphonate Use and AFF Risk

The likelihood of developing an AFF increases with prolonged exposure to bisphosphonate therapy [4, 11]. In a Southern Californian cohort (*n* = 196,129) of women 50 years of age or older receiving bisphosphonate therapy, 277 AFFs were detected following radiographical adjudication using the 2014 ASBMR case definition of AFFs (1.74 fractures per 10,000 patient-years) [11]. Following multivariable adjustment, compared with < 3 months of use, 3–5 years of bisphosphonate exposure was associated with an 8-fold increased risk of AFF (HR 8.86, 95% CI: 2.79, 28.20). The hazard ratio for 8 years or more of bisphosphonate use was 43.51 (95% CI: 13.70, 138.15). A recent Danish case cohort study utilising Danish National Healthcare records and blinded radiology review, found that compared with < 1 year of use, >3 to 5 years of bisphosphonate use was associated with an almost 4-fold increased risk of AFF (HR 3.94, 95% CI: 1.60, 9.65) and 5 to 7 years of bisphosphonate use was associated with a 7-fold increase in AFF risk (HR 7.29, 95% CI: 3.07, 17.30) [4]. The hazard ratio for 7 years or more of bisphosphonate use was 8.94 (95% CI: 3.92, 20.50). Interestingly, in this study “ever-use of bisphosphonate therapy” was added as a covariate to the multivariate models. Analyses performed without adjustment for any prior bisphosphonate use yielded hazard ratios more consistent with those reported by Black et al. (19.3 for 3–5 years of bisphosphonate use and 43.9 for > 7 years of use) [11]. Number needed to harm (NNH), and number needed to treat (NNT) were also evaluated. After > 5 years of anti-resorptive exposure, the NNH (for AFF development) was 1,424, whereas the NNT (for hip fracture prevention) was 56 in this Caucasian population. Extrapolated data over 10 years of anti-resorptive therapy suggested that the AFF NNH and the NNT for hip fractures began to align after a decade of use.

#### Drug Holidays and AFF Risk

AFF risk declines rapidly following anti-resorptive discontinuation [4, 11, 12]. Black and colleagues found that rates of AFFs declined following bisphosphonate cessation: 4.50 per 10,000 person-years among current users (including ≤ 3 months since discontinuation), 1.81 per 10,000 person-years at > 3 months to 15 months since discontinuation, and approximately 0.50 per 10,000 person-years at > 15 months after discontinuation [11]. Time since bisphosphonate discontinuation was associated with a 48% reduction in the risk of AFF at > 3 months to 15 months (HR vs. ≤ 3 months 0.52, 95% CI: 0.37, 0.72) and > 74% risk reduction in subsequent years. Bauer et al. also demonstrated a reduction in AFF risk following bisphosphonate discontinuation [4]. Compared to current use, time since bisphosphonate discontinuation saw a 47% reduction in the risk AFF at > 1 to 3 years (HR 0.53, 95% CI: 0.22, 1.28) and a 77% risk reduction after > 3 years (HR 0.23, 95% CI 0.05, 1.01). These findings accord with the previous reports of Schilcher and colleagues, where a 60–70% risk reduction in AFF occurred with every year passed since bisphosphonate discontinuation (multivariable-adjusted OR 0.29, 95% CI 0.26, 0.32 in female AFF cases and 0.37, 95% CI 0.30, 0.47 in male AFF cases) [12].

#### Ethnicity and AFF Risk

Asian ethnicity has emerged as an important risk factor for AFF [8, 9, 11, 13–15]. In a large population of Northern Californian women (*n* = 48,390), Asian women had an age-adjusted relative hazard ratio for AFF development of 8.5 compared with White women [9]. In another cohort of female bisphosphonate-users in Northern California, a large proportion (62.8%) of those who sustained an AFF were of Asian ethnicity [13]. Dhanekula et al. reported a four-fold higher proportion of Asian individuals sustained AFFs compared with typical femoral fractures (TFF) [16]. Black et al. reported an adjusted hazard ratio for AFF of 4.84 for Asian women compared with White women [11]. Similarly, we identified that Asians were 3.4 times more likely to sustain an AFF, with the highest risk seen in those of Southeast Asian ethnicity [8]. Our group also found that Asians, in particular those of Southeast Asian ethnicity, were 2- to- 3-times more likely to sustain an “earlier onset AFF” (an AFF sustained following ≤5 years of anti-resorptive use) compared with non-Asians [15]. These findings accord with a systematic review published in 2010 of 39 AFF case reports and case series, where individuals treated for ≤5 years were more likely to be of Asian origin [14].

Causes underlying the association between Asian ethnicity and AFF development remain an ongoing area of investigation. Favourable femoral geometry (smaller size, lateral bowing and smaller neck shaft angle) for AFF development in Asian individuals have been postulated to contribute. However, Dhanekula and colleagues recently found that, although Asian subjects had smaller femurs compared with non-Asians, femur neck shaft angle was similar in the Asian and non-Asian groups [16]. They purported that Asian AFF risk is independent of any ethnic specific differences in femoral geometry. Anti-resorptive drug metabolism, compliance, activity levels and genetic differences have also been thought to contribute.

**Fig. 2:**
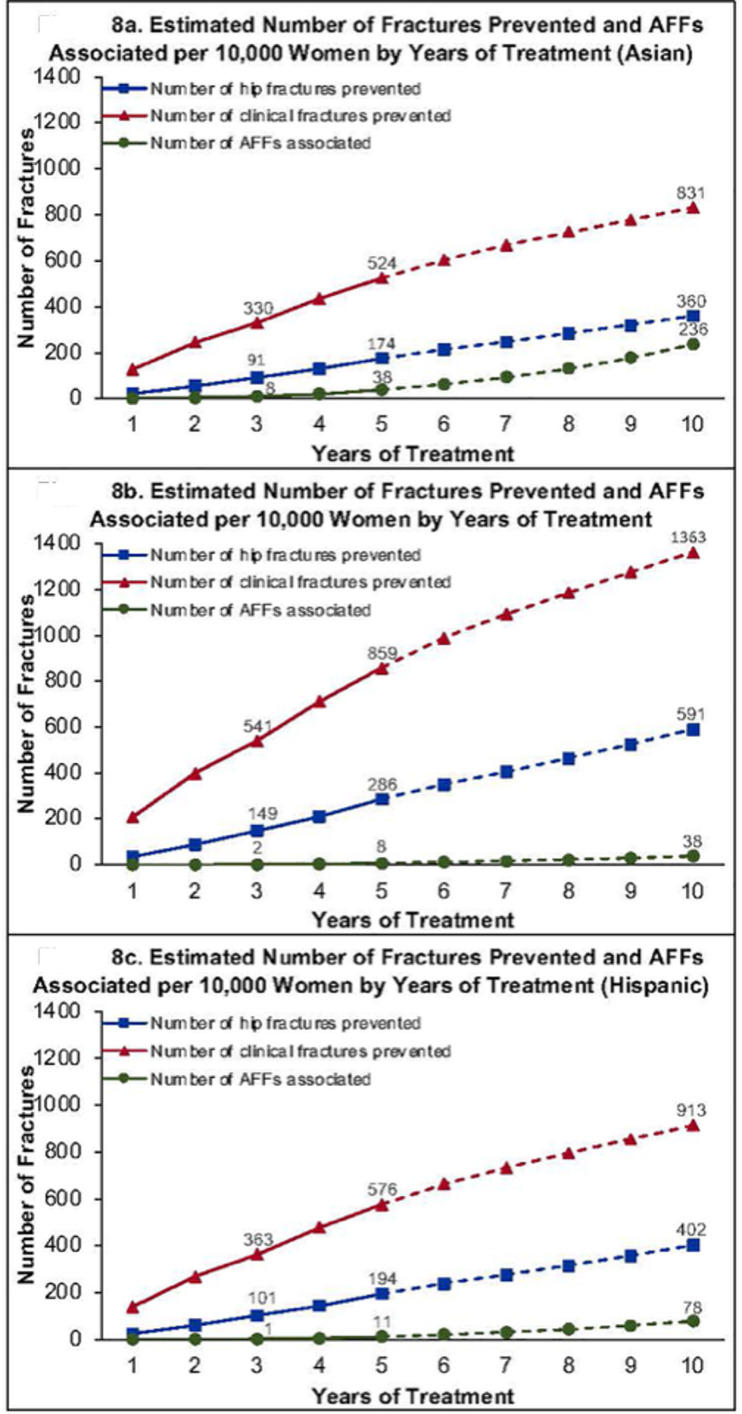
Number of hip and clinical fractures prevented compared to AFFs associated as a function of years of bisphosphonate treatment, by race (Asian (panel **A**), Caucasian (panel **B**) and Hispanic (panel **C**)). *(Courtesy of New Engl J Med).*

Finally, the accuracy of osteoporosis diagnoses based on DXA-derived bone density T-scores, fracture risk assessment and the optimal duration of anti-resorptive treatment in Asian individuals is an evolving and controversial area. Despite the International Society for Clinical Densitometry (ISCD) recommending the use of Caucasian (non-race adjusted) normative database for all ethnic groups for T-scores, studies have demonstrated that a significant proportion of Asian women are reclassified from osteoporosis to osteopenia when using an ethnic appropriate T-score [17, 18]. In addition, recent publications by working groups representing ASBMR and the International Osteoporosis Foundation demonstrate the vast spectrum of opinions as to whether race and ethnicity should be used in fracture risk assessment [19, 20]. Lastly, it is imperative to ground these considerations in the literature, consistently demonstrating that whilst Asian individuals appear more predisposed to AFFs, they exhibit a lower likelihood of sustaining osteoporotic fractures [11, 19, 21]. Black and colleagues demonstrated that among Whites, 3 years of bisphosphonate treatment prevented 149 hip fractures and 541 clinical fractures whilst only 2 AFFs were sustained [11] (Fig. 2). Whereas for Asians, after 3 years of treatment, 8 AFFs were reported whilst only 91 hip fractures and 330 clinical fractures were prevented. Importantly, distinct to Whites, after 10 years of treatment among Asians, the number of AFFs was only slightly less than the number of hip fractures prevented. The risk-benefit profile for anti-resorptive treatment therefore appears less favourable for Asian individuals than other ethnic groups.

#### Other Osteoporosis Treatments and AFF Risk

Data on AFF risk associated with other osteoporosis treatments is comparatively limited relative to bisphosphonate use. A recent review identified 31 AFFs in 22 patients treated with denosumab [22]. Of these, 11 patients (15 AFF) received denosumab 60 mg 6-monthly for osteoporosis, while 11 (16 AFF) received the higher dose of 120 mg monthly for metastatic Bone disease. Notably, 8 patients sustained AFFs without prior bisphosphonate use. AFFs have also been reported following raloxifene (*n* = 8), although treatment sequences were often unclear [22]. Four AFFs have occurred in patients treated with romosozumab, although two of these occurred during alendronate treatment following 12 months of romosozumab treatment [22–25]. Finally, a small number of AFFs have been associated with odanacatib, a cathepsin K inhibitor that decreases bone resorption with minimal suppression of bone formation [26]. Ten patients sustained 12 AFFs (adjudicated according to the ASBMR AFF case definition) among 8,043 treated with odanacatib, while no AFFs occurred in 8,028 placebo-treated participants. This suggests mechanisms other than suppressed bone turnover may contribute to AFF pathogenesis. Overall, the current evidence is insufficient to draw definitive conclusions about AFF risk associated with non-bisphosphonate treatments. While AFFs can occur with agents such as denosumab, raloxifene, romosozumab and odanacatib, the majority of data remains centred around bisphosphonate, predominantly alendronate, therapy.

#### Additional Clinical Risk Factors for AFF

Additional risk factors for AFF development include glucocorticoid use, proton-pump inhibitor (PPI) use, younger age, and increasing weight. Black and colleagues reported a greater than 2-fold increased risk of AFF in those having used glucocorticoids for ≥ 1 year compared to no use (HR 2.28, 95% CI: 1.52, 3.43) [11]. Bauer et al. found that AFF risk increased 7%, 22% and 40% after 1, 3 and 5 years of glucocorticoid treatment respectively [4]. Additional AFF risk factors reported by Black et al. include decreasing height (adjusted HR per 5-cm decrement 1.28, 95% CI: 1.15, 1.43), increasing weight (adjusted HR per 5-kg increment 1.15, 95% CI: 1.11, 1.19) and younger age (adjusted HR for 65 to 74 years vs. >85 years 2.76, 95% CI: 1.62, 4.72) [11]. A modest increased risk associated with PPI use was reported (HR 1.05 per year of use, 95% CI: 1.01, 1.08) by Bauer and colleagues [4].

#### Anti-resorptive Treatment

While AFFs have been reported with denosumab use [27–29], the majority of cases occur in the context of bisphosphonate therapy, particularly with oral bisphosphonates, and most notably with alendronate [2–4, 30]. This likely reflects, at least in part, global prescribing trends: alendronate is one of the most widely prescribed anti-resorptive therapies for osteoporosis worldwide [31, 32]. Assessing AFF risk associated with other treatment options (such as zoledronate, denosumab etc.), is complicated by two main limitations. Firstly, their use is less common, limiting the absolute event numbers to study. Secondly, patients may transition to these treatments following alendronate therapy, thereby making it difficult to determine whether AFF risk should be attributed to the current treatment or the preceding bisphosphonate exposure. Additional, variations in bisphosphonate pharmacology may influence AFF risk. Differences in hydroxyapatite binding and inhibitory potency for farnesyl diphosphate synthase (FPPS) can affect the potency and duration of action of bisphosphonates [33]. Capacity for binding to hydroxyapatite appears to lie in the following order: alendronate > etidronate > ibandronate > zoledronate > risedronate. Alendronate demonstrates strong hydroxyapatite binding and moderate inhibition of FPPS, with its higher bone uptake potentially explaining its greater and longer lasting suppression of bone turnover compared with lower-affinity analogues like risedronate. The extent to which these pharmacological effects influence variations in AFF risk among anti-resorptive treatments has yet to be elucidated.

#### Periprosthetic Fractures

The ASBMR Task Force 2010 and 2013 AFF case definitions specifically excluded periprosthetic atypical femoral fractures (PAFF) [2, 3]. However, a growing body of evidence suggests that certain periprosthetic femoral fractures (PFF) may exhibit atypical features, particularly in patients receiving long-term bisphosphonates [34, 35] A recent systematic review evaluated 17 patients from 12 studies who sustained PAFFs following hip arthroplasty [36]. All patients were female, and all were taking bisphosphonates at the time of atypical fracture, most frequently alendronate. Like AFFs, the majority (90%) of patients reported prodromal symptoms prior to fracture. All complete PAFFs radiographically demonstrated at least four ASBMR Task Force criteria for AFF (location, transverse fracture line, non-comminuted and medial spike) [3]. Makkar et al. reported a 1.3% prevalence of PAFFs amongst ICD-coded periprosthetic hip fractures in a cohort of American Veterans aged 50 years or older who had at least one filled anti-resorptive prescription (90% male cohort, mean age at fracture 76 ± 9.5 years) [37], whilst Leclerc et al. reported a frequency of PAFFs of 8.3% amongst PFFs (> 70% occurred in women) in Quebec City, Canada, over more than 9 years. Of the 11 PAFFs identified, six followed hip arthroplasty, three knee arthroplasties and two occurred in patients with both [38]. Whilst most PAFFs and PFFs following hip arthroplasties were Vancouver B1 fractures (fracture around the stem), PAFFs following knee arthroplasty tended to occur more proximally, away from the prosthesis. Hashimoto et al. prospectively reviewed cases of AFFs (*n* = 61) and included periprosthetic femoral fractures far from the prosthesis after total knee arthroplasty (TKA) without stem extension and defined them as periprosthetic AFFs (*n* = 4, 6.6%) [39]. Patients with hip arthroplasty were excluded. Biomechanical analysis using computed tomography (CT)-based finite element analysis (CT-FEA) revealed that the tensile stress distribution in one patient shifted from the distal to the proximal femoral diaphysis after TKA, corresponding to the fracture site. The authors hypothesised that both suppressed bone turnover from anti-resorptive treatment and mechanical stress due to femoral bowing may contribute to PAFF development.

Currently, the lack of a standardised definition hampers accurate estimation of PAFF incidence. Key questions remain: Should PAFFs be defined as occurring only after hip arthroplasty, knee arthroplasty or both? What distance from the prosthesis constitutes a PAFF? Is bisphosphonate exposure essential for diagnosis? Clinicians should be alert to atypical features of PFFs, particularly in patients on long-term anti-resorptive treatment.

#### Atypical Fractures at non-classical Sites

**Fig. 3:**
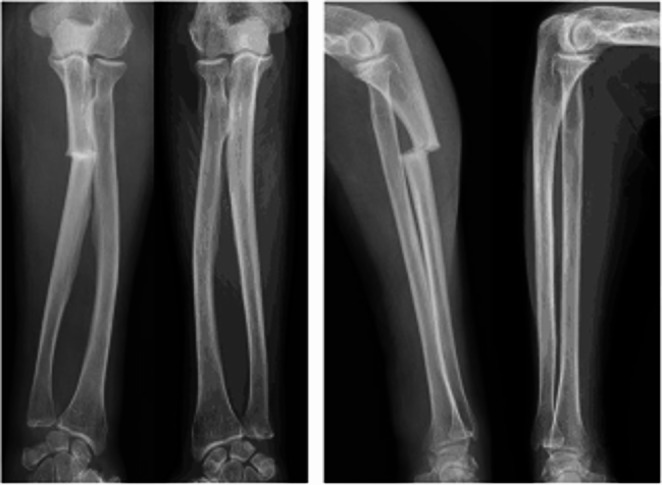
Preoperative radiography of bilateral forearm demonstrating left ulnar shaft fracture. *(Courtesy of BMC Musculoskelet Disord).*

At present, the ASBMR AFF case definition is limited to fractures located along the femoral diaphysis, originating at the lateral cortex [3]. Increasingly, case reports and case series have reported fractures with features like AFFs occurring at sites distinct from the femur [40] (Fig. 3). We recently published a systematic review of all cases of atypical fractures in patients (aged > 18 years) receiving long-term anti-resorptive therapy (> 3 years) and identified 149 cases of atypical fractures [41]. The ulna, followed by the tibia, were the most frequent fracture sites. All patients were taking anti-resorptive therapy prior to/at the time of fracture, most commonly alendronate. Like the ASBMR AFF case definition, the most common atypical fracture characteristics were the transverse fracture line (95%), non-comminution (98%) and cortical beaking (67%). Fractures at non-classical sites with atypical features should raise suspicion and prompt consideration of anti-resorptive cessation, limited weight bearing and surgical management if appropriate. An update of the ASBMR case definition may be timely.

### Update on Pathophysiology

#### Genetic Susceptibility

The development of AFFs in families, bisphosphonate-naïve individuals and individuals with monogenic bone diseases have raised suspicion of a genetic predisposition underlying AFF development [42, 43]. Two recent systematic reviews highlighted the presence of AFFs in monogenic bone diseases: hypophosphatasia, X-linked hypophosphataemia (XLH), pycnodysostosis, osteopetrosis, osteoporosis pseudoglioma syndrome (OPPG), osteogenesis imperfecta (OI) and X-linked osteoporosis [6, 7]. Several genetic studies have been completed to elucidate whether particular genetic pathogenic variants contribute to AFF development. While the methodology of each study is quite distinct and heterogenous, they generally undertake one or both of; a candidate gene-based approach (typically using variants previously implicated in AFF development/bone mineralisation) and an unbiased variant filtering approach.

Firstly, examining the studies that have utilised a candidate gene-based approach, contrasting results have been reported. Zhou et al. evaluated the prevalence of monogenic Bone disorders in a Dutch cohort of 60 AFF patients recruited from two specialist bone centres [43]. Nine patients (15%) were found to have a (likely) pathogenic variant in either *PLS3*,* COL1A2*,* LRP5*,* ALPL or TCIRG1.* Marini et al. performed next generation sequencing (NGS) analysis using a panel of 76 genes involved in bone Mineralisation in a cohort of 25 unrelated patients with at least one AFF [44]. They identified a rare variant in *SLC34A1* or *SLC9A3R1* in over a third (32%) of their patient cohort (4 cases bore a *SLC34A1* variant and 4 cases bore a *SLC9A3R1* variant). Other rare genetic variants identified included *BMPR1B*,* CYP27B1*,* FBN1*,* MEPE*,* PIGO* and *PHOSPHO1* gene, each in a single AFF case. Peris et al. performed Sanger sequencing of the *ALPL*, *GGPS1* and *CYP1A1* genes in 17 women with AFF [45]. Three patients (18%) had an identified variant in *ALPL* or *CYP1A1*. Furukawa et al. identified an increased frequency of rare variants of *ENPP1* in Japanese AFF cases (*n* = 42) compared with controls [7 cases (8.3%) vs. 180 controls (1.9%), *p* = 0.0012, corrected *p* = 0.0155] [46]. Of note, this is one of the few genetic studies performed in individuals of Asian ethnicity, for whom the risk of AFF is increased.

In contrast, Zhou et al. studied two Singaporean families [mother (1-a) and daughter (1-b), and two sisters (2-a and 2-b)] [47], all who sustained bisphosphonate-associated AFF. Of note, DNA for 1-a was not available. Utilising a candidate gene-based approach, no rare variants associated with AFF were shared between the three cases (1-b, 2-a, 2-b). A rare variant in *TMEM25*, shared between the two sisters, was isolated, as was a rare *PLOD2* variant in the daughter (1-b) (however this was not shared with the sisters). Kharazmi et al. undertook candidate gene analyses (29 genes previously implicated in AFF development) in a cohort of 51 patients with AFF and found no significant association [48]. Del Real et al. performed exome sequencing in 13 AFF patients (utilising a large candidate gene list of 457 genes related to skeletal homeostasis) [49]. A pathogenic variant in *ALPL* was only identified in a single AFF case. Further work identified more variants in AFF cases than osteoporotic controls without AFF, particularly in the genes *ACAN*,* AKAP13*,* ARHGEF3*,* P4HB*,* PITX2* and *SUCO*. Zhou et al. performed a candidate gene analysis (203 genes) on 139 European AFF cases and identified only *PLOD2* as a potentially significant damaging variant [50]. No other genetic variants were isolated, particularly those previously identified by other studies such as *ENPP1*,* SLC34A1*,* SLC9A3R1*,* GGPS1* or *CYP1A1* [42, 44, 46]. Similarly, Zhou et al.. did not identify a pathogenic variant associated with monogenic bone diseases in three siblings with bisphosphonate associated AFFs [51].

Unbiased variant filtering has been utilised to identify genetic variants of interest. Focusing on genetic studies undertaken in families affected by AFFs, Zhou et al. identified 13 potential pathogenic variants (*VIPR1*,* LOXL4*,* ROBO3*,* SLC12A2*,* APC*,* ECSIT*,* TDRD6*,* SCN4A*,* ABL2*,* TMEM138*,* MLLT4*,* RNF213*) shared by three siblings with bisphosphonate-associated AFFs [51]. Particular emphasis was placed on lysyl oxidase like 4 (*LOXL4*) due to its function in formation of collagen crosslinks. The same group identified seven potential genetic variants (*EPHA10*,* SORCS1*,* GSX2*,* PARP2*,* C19orf60*,* APH1B*,* PHYHD1)* shared by Singaporean sisters (2-a and 2-b) [47]. None of these variants were found in the daughter (1-b) of the first Singaporean family. Another study identified variants in *GGPPS* and *CYP1A1* shared between three sisters with AFFs [42]. However, further work, including three additional unrelated individuals with AFFs, identified no shared genetic variants between the six individuals [52]. Unrelated individuals with AFF have also been studied. Zhou et al. identified 10 potential genetic associations in a large cohort of 139 AFF cases, with added weight placed on *XRN2*,* PLOD2* and *SORD* genes [50]. Garcia-Giralt et al. identified 132 potential genes of interest in 12 individuals with AFFs [53]. Twelve of these genes were known to be implicated in bone and/or AFF pathophysiology. Perez-Nunez et al. identified 21 potential novel variants with a trend for association with AFFs (unadjusted *p* < 0.0025) in 13 women with AFFs [54]. Finally, Kharazmi et al. did not identify any potential genetic variants when AFF cases (*n* = 51) were compared with bisphosphonate-treated controls [48].

Genetic heterogeneity of AFF development appears evident. Many potential genetic variants have been implicated. An area of ongoing interest is that of genetic susceptibility in individuals of Asian ethnicity, a population with established increased AFF risk [8, 9, 11]. Few genetic studies have evaluated this population [46, 47]. Further work is required to elucidate whether genetic predisposition underlies the difference in AFF risk between Asian and non-Asian individuals.

#### Lower Limb Geometry

The revised ASBMR 2013 AFF case definition stipulates that an AFF can be located anywhere along the femoral diaphysis (just distal to the lesser trochanter to just proximal to the supracondylar flare). However, the majority of AFFs occur at two dichotomous locations: the subtrochanteric and diaphyseal regions [3, 55]. Femoral geometry, strain patterns and mechanical loading have been postulated to contribute to this difference.

Multiple reports have found that individuals who sustain AFFs have greater lateral and/or anterior femoral bowing compared with those who sustain typical femoral fractures [56–58]. In addition, increased femoral bowing is associated with a more distal AFF location [58–61]. AFFs occur at areas of maximum tensile stress, and the location appears strongly influenced by the bowing of the femoral shaft [59]. Using a CT-FEA model, Hwang and colleagues recently demonstrated that indeed the weakest femoral point coincided with AFF location [62]. In addition, the weakest point was in the femoral shaft area in bowed femurs, in comparison to the subtrochanteric region in straight femurs. The maximum tensile stress loading area moved to the distal femur based on varus alignment. Decreased body weight and increased age have also been associated with a diaphyseal fracture pattern [60]. A possible risk factor for subtrochanteric AFFs has been postulated by several groups [58, 59]. A smaller, or more acute, femoral neck-shaft angle has been identified in subtrochanteric AFFs compared with diaphyseal AFFs [58, 59]. The low bone strength due to a thin lateral cortex has been speculated to contribute to the development of subtrochanteric AFFs [59]. The impact of other geometric parameters, e.g. femoral head size, femoral neck offset, on AFF development and location remains equivocal [16].

Certain femoral geometric parameters have been postulated to partially explain the elevated AFF risk in Asians. Several reports have demonstrated that Asian subjects have greater femoral bowing compared with non-Asian peers [56, 63, 64]. However, a study challenged our understanding of the relationship between ethnicity, femoral bowing and fracture location [64]. Schilcher et al. compared Singaporean and Swedish AFF subjects. An acceptable hypothesis would be that the Singaporean cohort would have greater femoral bowing compared with the Swedish cohort, and therefore an increased number of diaphyseal AFFs. Instead, counterintuitively, the group demonstrated more AFFs were subtrochanteric in the Singaporean cohort, whilst more were diaphyseal in Location in the Swedish cohort. Of note, the median age of the Swedish AFF cohort was almost 10 years older than that of the Singaporean cohort (78 years vs. 69 years). This difference may have contributed to these unexpected findings. Alternatively, variations within the Asian race itself may contribute to differences in AFF location.

#### The Concept of Bone Disorganisation

Bone disorganisation, simply meaning bone that is inappropriately arranged or located, is a novel concept postulated to contribute to AFF pathogenesis [65]. Contributory elements include prolonged use of anti-resorptive treatments, glucocorticoid therapy and an underlying monogenic bone disorder. Zebaze et al. have also proposed a method by which bone disorganisation can be detected and quantified in vivo [66]. Using a femoral radiograph, a unique Disorganisation Quantifier APP (ALIGNOGRAM) highlights the presence and extent of bone disorganisation along the femur. Further work is required to ascertain the clinical applicability of the ALIGNOGRAM in AFF detection and predisposition.

#### Screening for AFFs

Despite distinct radiological features from typical femur fractures (TFFs), AFFs remain under detected. Retrospective audits demonstrate low rates of AFF classification in radiology reports at large health services [67, 68]. Incomplete AFFs may have subtle x-ray changes that are often missed by untrained clinicians. These detection errors may result in delayed or suboptimal treatment with resultant poor healing, progression of incomplete AFFs or the development of contralateral AFFs. To improve AFF detection, numerous studies have explored novel methods to screen for AFFs and discriminate AFFs from TFFs.

Crouch et al. developed a novel Sydney AFF Score to discriminate AFF from TFF cases using quantitative, measurable parameters [69]. This simple tool uses three dichotomised independent predictors in a female cohort with one point for the following parameters: age $\:\le\:$80 years; femoral neck width <37 mm; and lateral cortical width at lesser trochanter $\:\ge\:$5 mm. A score $\:\ge\:$2 demonstrated 73.3% sensitivity and 69.6% specificity for AFF on external validation. The retrospective nature of the study precludes its use as a prospective risk score for the development of AFF in long term bisphosphonate users, however it describes high-risk demographic and femoral geometric features associated with AFFs.

**Fig. 4:**
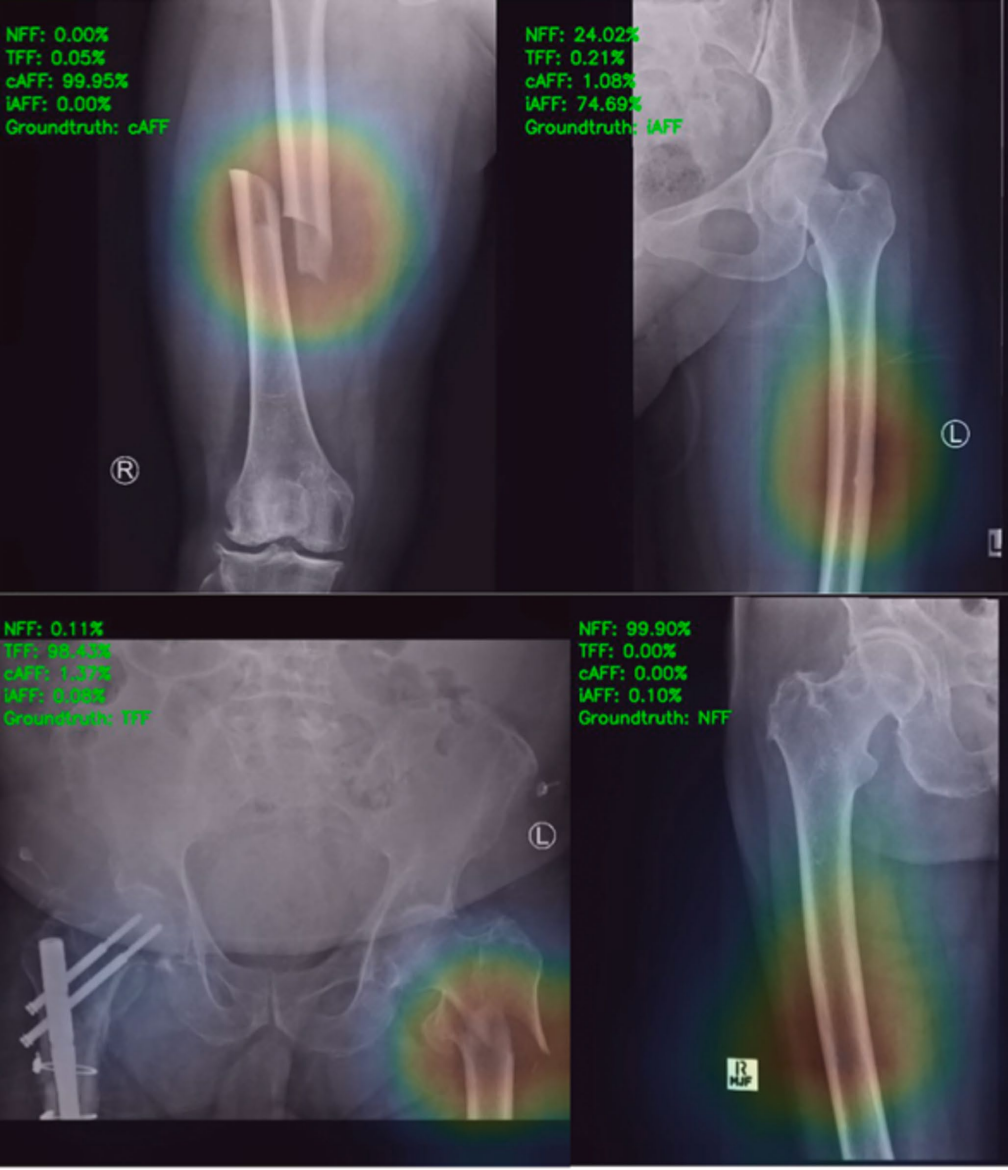
Examples of model predictions and computerized activation maps. *(Courtesy of Bone).*

Few studies have explored artificial intelligence-based computer vision technology, using convolutional neural networks (CNNs), to classify AFFs from radiographs. Zdolsek et al. trained several deep neural network structures on 433 complete AFFs and 549 TFFs [70]. They found the CNN ResNet-50 to have the highest diagnostic accuracy in classifying complete AFFs from TFFs (AUC 0.94). Further work from this group showed that fusion of radiographs with clinical data further enhanced the power to detect AFFs, with sensitivity increasing from 0.796 to 0.903 [71]. Kim et al. trained a model on 100 incomplete AFFs and 950 normal femur radiographs, with their model demonstrating high diagnostic performance (AUC exceeding 0.99 on internal validation testing) [72]. Recently, we published on AFFnet, a model that can classify incomplete AFFs, complete AFFs, TFFs, and normal femur radiographs [73] (Fig. 4). Utilising 2,015 radiographs for training, the model was internally tested and externally validated. High diagnostic performance (AUC > 0.97) was demonstrated, and the sensitivity in detecting incomplete AFFs was 82%. These preliminary studies show promise in the development of an automated AFF detection software that can screen radiographs and alert clinicians to fracture features concerning for AFFs, however further work to validate these models in the real-world setting is necessary.

Serious consequences of AFFs may be prevented if subtle AFF changes, such as cortical beaking, or incomplete AFFs are detected early. Full-length image of the femur (FFI) by single energy (SE) x-rays can detect a spectrum of femoral changes that may represent an incomplete AFF, such as focal cortical thickening at the lateral femur with or without a lucent fracture line. This additional DXA software scans bilateral femur at the time of a DXA exam, with very Low radiation exposure. In 2019, the ISCD published an official position statement on the detection of AFFs using FFIs [74]. Following a cumulative exposure of 3 or more years of bisphosphonate or denosumab therapy, clinicians could consider bilateral FFI for detecting abnormalities in the spectrum of AFF, especially in those on glucocorticoid therapy. However, a recent study by Chaaya et al. reported a low yield for FFI screening of AFFs [75]. In this audit of 948 cases with > 3 years of anti-resorptive therapy at a large health service in Lebanon, FFI identified 18 cases with femoral changes in the spectrum of AFF (1.9%). However, only one case had a confirmed incomplete AFF by subsequent imaging. The yield of FFI screening in a higher AFF risk population (glucocorticoid exposure, Asian ethnicity, monogenic bone disorders) and the optimal protocol for surveillance is yet to be examined.

### Management

#### Surgical Treatment

The mainstay of surgical treatment for complete AFFs is closed intramedullary nail [2, 76–79]. Koh et al. systematically reviewed the treatment of both complete and incomplete AFFs [80]. Most complete fractures were managed with intramedullary nailing, rather than plating (locking compression plate, dynamic hip screw, dynamic condylar screw and angled blade plate). More AFFs treated with plate fixation required revision surgery compared with intramedullary nailing (31.3% vs. 12.9%, *p* < 0.01).

Following complete AFF, it is imperative to formally image the contralateral femur due to the increased risk of subsequent contralateral AFF [3]. Possible imaging modalities include DXA, plain radiography, magnetic resonance imaging (MRI), CT and radionuclide bone scan [3]. Shim et al. proposed a treatment algorithm for suspected AFFs [79]. The decision to prophylactically nail an incomplete AFF focuses on alleviating pain, preventing progression to a complete AFF, and avoiding the healing challenges and implant complications associated with a complete fracture [79]. Prophylactic intramedullary nailing of incomplete AFFs has been predominantly recommended for individuals with thigh pain [3, 80], however some authors have also recommended it for incomplete AFFs with the “dreaded black line”, varus femoral bowing or a failure to improve following two or three months of conservative management [79]. Supportive of these recommendations, Koh et al. reported that of 159 incomplete AFFs, only 72 (45%) healed after non-operative treatment. However, 32 (30%) fractures progressed to a complete fracture, 43 fractures (27%) required surgery due to pain refractory to conservative management, and 12 fractures (8%) had not healed during follow-up. On the other hand, of the incomplete fractures treated with prophylactic surgery, 97% (106/109 fractures) healed without requirement for revision surgery [80]. In addition, Min et al. proposed a scoring system to assist in identifying impending complete AFFs amongst incomplete AFFs [81]. They recommended that an incomplete AFF with a score < 8 could be treated conservatively, whilst lesions with scores ≥ 8 require prophylactic fixation. Elements of the score included subtrochanteric location, pain, an intact contralateral femur and radiolucent line > 50% of the diameter of the femur. Recently, Jiang et al. utilised Markov modelling to determine the cost-effectiveness of contralateral prophylactic fixation after an initial AFF [82]. They reported that prophylactic fixation is cost-effective amongst patients aged 60 to 89 years of age with more than one risk factor for an AFF such as Asian ethnicity, prodromal pain, varus proximal femur geometry, femoral bowing and/or radiographic changes such as periosteal beaking and a transverse radiolucent line, the “dreaded black line”.

#### Medical Treatment

After an AFF, potent anti-resorptive therapy should be discontinued, vitamin D and calcium supplementation considered, surgical treatment completed if indicated, weight-bearing restricted, imaging of the contralateral femur performed, and the underlying osteoporosis addressed [3]. Echoing these recommendations, van de Laarschot et al. published their systematic review of the medical management after AFF, and recommendations from the European Calcified Tissue Society [22]. A clear decision tree was proposed to summarise their expert recommendations following AFF. In addition, Adler recently proposed their management scheme for the treatment of osteoporosis following AFF [83]. The use of teriparatide following both incomplete and complete AFFs is a current area of interest. Within the limits of the existing evidence, observational data may suggest that teriparatide is beneficial to the healing time of surgically managed AFF [22]. Salamah et al. systematically reviewed the current literature and found that teriparatide increased the incidence of early bone healing and decreased the time to bone union in surgically managed AFFs [84]. No significant differences were seen with complete bone healing, non-union and progression of incomplete AFF to complete AFF [84]. Similarly, Byun et al. found that teriparatide may benefit fracture healing, shortening healing time and reducing the rate of delayed union [85]. Data do not suggest that teriparatide is beneficial in the healing of conservatively managed incomplete AFFs. Duration of teriparatide used solely to enhance fracture healing may be 3 to 6 months. For individuals at high fragility fracture risk, teriparatide may be used as an ongoing osteoporotic treatment. Van de Laarschot et al. recommend that, for patients receiving teriparatide for the ongoing management of their underlying skeletal fragility, to closely monitor bone turnover markers, and consider treatment with a selective estrogen receptor modulator (SERM), romosozumab, calcitonin, tibolone, estrogens, denosumab/ bisphosphonates (based on bilaterality of surgical intervention), if bone markers start to rise or when bone mineral density begins to decrease following teriparatide treatment [22].

Introducing a drug holiday, a temporary pause in anti-resorptive therapy, has proven effective in lowering the risk of AFF [4, 11, 86]. Decisions about the long-term use of anti-resorptive therapy, including the consideration of a drug holiday, should be tailored to each individual patient [87]. Clinicians should weigh the benefits of ongoing treatment against the possible side effects of treatment continuation, and fracture risk during a holiday period. Furthermore, the type of bisphosphonate may play a role in determining the approach to a drug holiday. Mortality following oral bisphosphonate drug holiday was assessed by Leung et al. [88]: 365 patients presenting with hip fractures were analysed. Patients who had discontinued risedronate for 1 and 2 years had a higher post fracture mortality compared with those who had discontinued alendronate for 1 and 2 years (HR 2.37; 95% CI, 1.24, 4.53 and HR 3.08; 95% CI, 1.48, 6.41 vs. HR 0.59; 95% CI, 0.29, 1.18 and HR 1.05; 95% CI 0.57, 1.93). This finding may be influenced by the greater hydroxyapatite binding potency of alendronate compared with risedronate [33].

## Conclusion

This review summarises relevant updates on the epidemiology, pathogenesis and management of AFFs since 2018 [89]. AFFs remain a rare side effect of anti-resorptive treatment. Evidence demonstrates that the risk of developing an AFF increases with extended anti-resorptive therapy use. A subset of individuals that appear at increased risk of AFF development is those of Asian ethnicity. Important questions for future study include, (1) Are there different risk profiles within the Asian race? (2) Why is the risk higher in those of Asian ethnicity? (3) Are there genetic differences that underly the ethnic difference? and (4) Should treatment recommendations be different for Asian individuals?

Several areas of future work remain. A review and update of the current ASBMR AFF case definition to include atypical fractures at sites other than the femur and periprosthetic AFFs may be timely. Secondly, further genetic studies, especially in individuals of Asian ethnicity, are required to elucidate whether a genetic predisposition is implicated in AFF development. Lastly, the emerging development and application of screening tools, AFF risk scores and artificial intelligence technology for classifying and diagnosing AFFs are crucial, given persistently low detection rates.

## Data Availability

No datasets were generated or analysed during the current study.
